# Regulated synthesis of Zr-metal–organic frameworks with variable hole size and its influence on the performance of novel MOF-based heterogeneous amino acid–thiourea catalysts†

**DOI:** 10.1039/d2ra03747e

**Published:** 2022-08-03

**Authors:** Junfeng Zhu, Xiaorong Meng, Wen Liu, Yabing Qi, Siyi Jin, Shanshan Huo

**Affiliations:** College of Chemistry and Chemical Engineering, Xi'an University of Architecture and Technology Xi'an 710055 China mxr5@163.com; Research Institute of Membrane Separation Technology of Shaanxi Province Co., Ltd Xi'an 710055 China

## Abstract

We present an efficient and easy synthesis method for incorporating organocatalytic moieties into Zr-metal organic frameworks (Zr-MOFs). The catalytic activity and selectivity of the new chiral catalysts were improved by adjusting the aperture of the MOF cavities. The hole size of the Zr-MOF was modulated by adding acid and replacing bridge ligands during synthesis. The difunctional chiral units of amino acid–thiourea are anchored onto the Zr-MOF by a mild synthesis method from an isothiocyanate intermediate which could effectively avoid the racemization of chiral moieties in the synthesis process. By means of specific surface area measurement (BET), scanning electron microscopy (SEM) and powder X-ray Diffraction (PXRD), it was confirmed that Zr-MOFs with different pore sizes were synthesized without breaking the basic octahedral structure of the MOF. Finally, good yields (up to 83%) and ee values (up to 73%) were achieved with the new heterogeneous catalysts in 48 hours for the aldol reaction of 4-nitrobenzaldehyde with acetone. By contrast, using the catalyst support without modulating the synthesis, the yield (30%) and the ee-value (26%) were both low. Experiments have confirmed the important influence on the reaction selectivity of providing a suitable reaction environment by controlling the aperture of MOF cavities.

## Abstract 2

A MOF-NH_2_ material was modified with amino amide derivatives to construct heterogeneous phase MOF catalysts. The catalystsdisplayed efficient catalytic activity in asymmetric aldol reactions.

## Graphical abstract

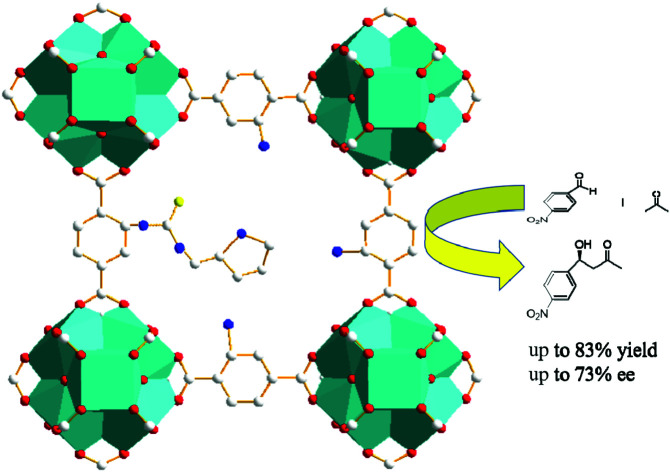

## Introduction

Metal–organic frameworks (MOFs), which constitute a new class of functional hybrid nanoporous materials,^1^ have potential applications in photocatalysis,^2^ sensing,^3^ gas storage,^4^ and separation.^5^ In addition, porous functionalized MOFs for high value-added applications have been increasing rapidly in domains such as enantioselective heterogeneous catalysis^6,7^ and chiral separation.^8^ The characteristics and applications of MOFs could be regulated easily by adjusting the ligands, metal ions or clusters, solvent, additives and synthesis conditions.^6,9–12^ In particular, some of these MOFs could provide a suitable reaction environment through control of the aperture of the MOF cavities.^13^

Amino acid and its derivatives, which are practical organocatalysts,^14^ have been widely used for catalyzing asymmetry organic reactions, such as aldol, Michael addition and Mannich reaction.^15–18^ Given the existence of regularly ordered chiral functionalities to provide high enantioselectivity, amino acids are anchored into the MOF cavities to generate a heterogeneous catalyst. Amino acids (particularly proline) could be grafted in MOF cavities either by self-assembly using functionalized ligands^19^ or by post-synthetic functionalization starting from easily accessible amino-containing frameworks.^20^ However, when the self-assembly process involves a thermal treatment such as the removal of the thermolabile Boc-group on proline at a high temperature of over 100 °C,^21^ the racemization of chiral center often occurs. Thus, the enantioselective properties of the catalyst are often poor. In addition, post functionalization has a low grafting rate in MOF with small cavities. Therefore, it is often detrimental to the catalytic effect of catalyst or the quality of the final material. In recent years, proline-functionalized MOFs are built on the framework of MIL,^22^ UiO,^23^ IRMOF,^24^ DUT^25^ and PCN,^26^ but an unsatisfactory enantioselectivity is obtained in heterogeneous asymmetric reaction.^27^

Organic chemists have discovered that a chiral catalyst with hydrogen-bond donor motifs associated with complementary functional frameworks such as thiourea often has high enantioselectivity,^28,29^ because of the H-bond donor site flanked by sites for secondary interaction with substrates. In our work, proline–thiourea motifs have been first covalently grafted inside MOF cavities by a mild synthesis method, to design a chiral heterogeneous catalyst. Zr-MOF as the starting platform has been investigated. UiO-66-NH_2_ is based on Zr_6_O_6_ clusters linked by 2-aminoterephthalate.^30^ Give its good acid and alkali resistance and thermostability, it is very suitable for an organic catalytic support. However, UiO-66-NH_2_ has difficulty in grafting, because of smaller accessible cavities. Canivet and co-workers reported the condensation of proline and a 2-aminoherephthalate ligand in UiO-66 under a microwave-assisted method, but the graft yield was less than 10%. They hypothesized that the size of the UiO-66 pore aperture is the main problem, because the graft yield could be more than 60% during the condensation of proline and the 2-aminotherephthalate ligand in Al-MIL-101, which has a larger cavity size.^31^

**Scheme 1:**
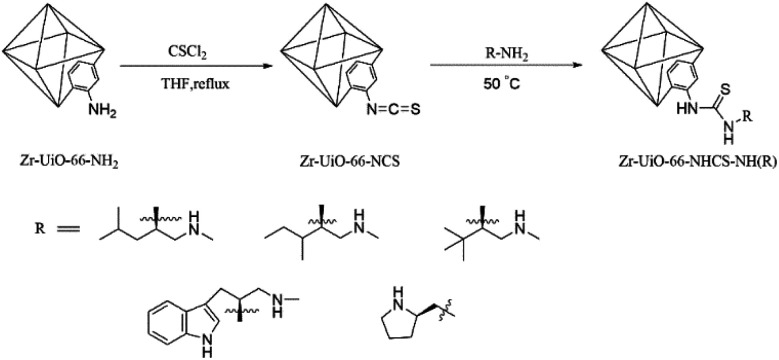
Post-synthetic process of Zr-UiO-66-NH_2_.

In addressing these problems, a new method of introducing chiral skeletons is applied in this paper. The amino of Zr-UiO-66-NH_2_ is initially treated with sulfur phosgene to generate high reactive isothiocyanate. Then, the amine obtained by reducing natural amino acids anchored to the MOF carrier with high yields through a reaction between the 1° amino of the amine acquired by the reduction and the isothiocyanate group on the MOF (Scheme 1), and a H-bond donor (thiourea) was formed simultaneously. The difunctional chiral units could improve the activity and enantioselectivity of the new catalyst. The effect of the aperture of MOF cavities was studied to further improve the catalyst performance. The aperture of MOF cavities was regulated by adding acid additive and changing the size of bridge ligand during the synthesis of MOF. The detailed synthesis of Zr-UiO-66-NHCS-NH(R) was given in Fig. S1–S5.†

## Experimental section

### Materials

Zirconium tetrachloride (ZrCl_4_, 99.5%), 2-aminoterephthalic acid (H_2_BDC, 99%) and *N*,*N*′-dimethylformamide (DMF, 99.5%) were obtained from Shanghai Aladdin Bio-Chem Technology Co., Ltd. Leucine, isoleucine, *t*-leucine, tryptophane and proline were purchased from China National Pharmaceutical Group Corporation. Tetrahydrofuran (THF, AR), concentrated hydrochloric acid (HCl, AR) and acetic acid (CH_3_COOH, AR) were purchased from Shanghai McLean Biochemical Technology Co., Ltd. All reactions were carried out in anhydrous solvents. All ligands, chemicals, and solvents were purchased from Aladdin and McLean Chemical Industry and used without further purification.

### Characterization

Fourier Transform Infrared Spectroscopy (FT-IR) spectra of samples in the mid-infrared region was conducted using a Nicolet iS5 infrared spectrometer of Thermo Company in the United States. The detected wavenumber range was from 500 to 4000 cm^−1^, and the resolution was 4 cm^−1^. Furthermore, KBr tablet pressing was used for testing. X-ray diffraction (PXRD) were obtained by diffraction data using a MiniFlex600 powder X-ray diffractometer by Cu-Kα radiation in the 2*θ* range of 5°–90° in 0.03° steps. Approximately 15 mg of microcrystalline MOF samples was dried at 100 °C for at least 2 h before analysis. Nuclear magnetic resonance (^1^H NMR) were recorded using a Brüker 500 MHz spectrometer. Chemical shifts were reported in parts per million (ppm) in accordance with the appropriate solvent peak. Prior to NMR analysis, MOF samples were dissolved in a NaOH-D_2_O solution. N_2_ adsorption and desorption tests was performed using an APSP 2460 physical adsorption apparatus from Micromeritics USA. The samples were vacuum degassed at 120 °C for 12 h before starting the measurement. The degassed samples were tested for adsorption and desorption at a liquid nitrogen temperature (77 K) with nitrogen as the adsorbate. The ee-values of the compounds were obtained by Shimadzu LC-20 A chromatograph. The peaks for the enantiomer of each aldol product in chiral HPLC were determined by comparing with racemic samples. Scanning Electron Microscope (SEM) was conducted on a Gemini SEM 500 electron microscope from Zeiss, Germany, with a test voltage of 5 to 10 KV and a working distance of 1 to 10 mm. Before the test, the sample was fixed on the test bench using a conductive tape for surface gold spraying treatment, and then the sample was sent to the observation chamber and evacuated for characterization.

### Synthesis of Zr-UiO-66-NH_2_

Zr-UiO-66-NH_2_ was synthesized by a solvothermal method: First dissolve zirconium tetrachloride and 2-aminoterephthalic acid were dissolved 480 times in a molar amount of DMF After sonication, the solution was transferred to a Teflon autoclave and reacted at 120 °C for 24 h. After the reaction was completed, it was cooled to room temperature, and the reaction product was filtered and washed with DMF to remove unreacted ligands. In addition, the solid was collected by centrifugation. The DMF of the exchange product was soaked in methanol (3 × 30 mL). Vacuum drying at 60 °C yielded a pale yellow solid.

### Synthesis of Zr-UiO-67-NH_2_

Zr-UiO-67-NH_2_ was synthesized by a solvothermal method: First dissolve zirconium tetrachloride and 2-amino-[1,1′-biphenyl]-4,4′-dicarboxylic acid was dissolved 480 times in a molar amount of DMF. After sonication, the solution was transferred to a Teflon autoclave and reacted at 120 °C for 24 h. After the reaction was completed, it was cooled to room temperature, and the reaction product was filtered and washed with DMF to remove unreacted ligands. In addition, the solid was collected by centrifugation. The DMF of the exchange product was soaked in methanol (3 × 30 mL). Vacuum drying at 60 °C yielded a pale yellow solid.

### Synthesis of catalyst

In a 50 mL glass vial, the desired amount of MOF-NH_2_ (*ca.* 1.0 mmol –NH_2_), in 20 mL of anhydrous THF. The suspension was stirred at 25 °C for ten minutes. Then 5.0 mmol CSCl_2_ (0.4 mL) was added and the suspension was allowed to react under tirring for twenty-four hours at 66 °C. The resulting suspension was cooled to room temperature and centrifuged. The solid was washed with THF (3 × 10 mL). Then it was suspended in THF (20 mL) again, and 1.2 mmol pyrrolidin-2-ylmethanamine (R) was added. The suspension was stirred for another twenty-four hours at 50 °C. Then it was centrifuged and the solid was washed with THF (3 × 10 mL) to give the desired product as a fine yellow powder after drying under vacuum at 50 °C.

### Catalysis of aldol reaction

In a typical catalytic trial, catalysts were suspended in a solution of *p*-nitro-benzaldehyde in acetone at room temperature. After stirring, the suspension was separated by centrifugation and the liquid phase was purified by chromatographic column. Then white solid was afforded after the solvent was removed by rotary evaporation under reduced pressure. The enantiomeric excess (ee) were analysed by HPLC.

## Results and discussion

### Synthesis and characterization of catalyst

**Fig. 1:**
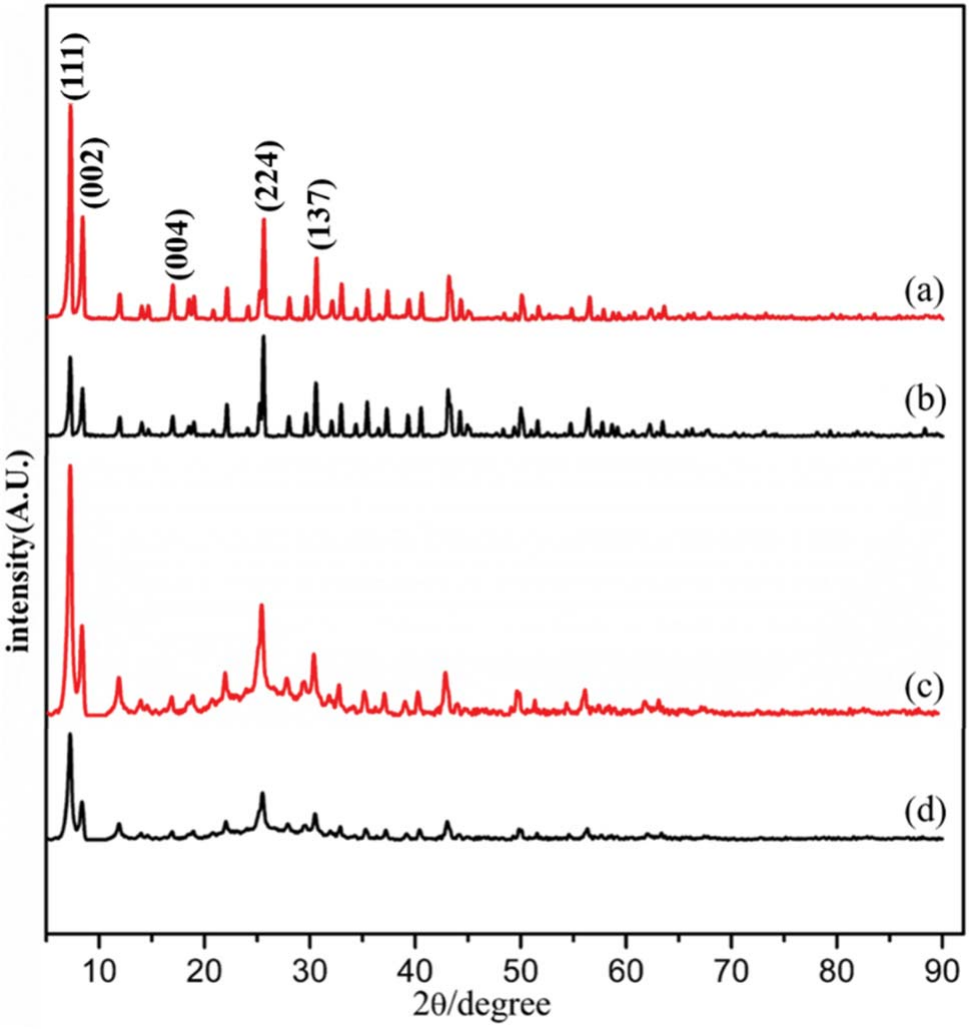
PXRD of (a) Zr-UiO-66-NH_2_ modulated by 90 eq HAc and 1 eq HCl, (b) Zr-UiO-66-NH_2_ without acid modulation, (c) Zr-UiO-67-NH_2_ modulated by 90 eq HAc and 1 eq HCl, (d) Zr-UiO-67-NH_2_ without acid modulation.

**Fig. 2:**
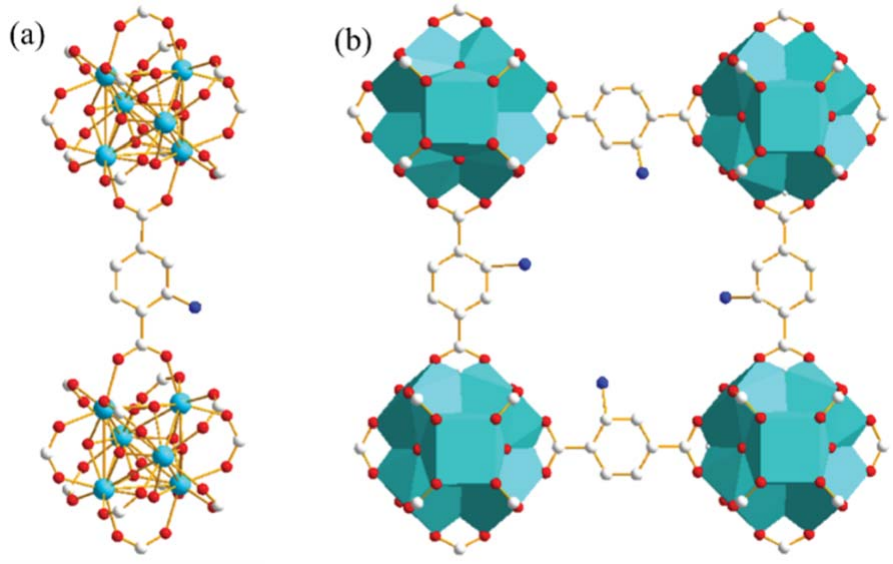
Structure of Zr-UiO-66-NH_2_ determined by XRD. (a) The ligand was BDC–NH_2_, and the was node Zr_6_O_4_(OH)_4_. Red = O; white = C; blue = N; green = Zr. (b) Typical cubic repeating unit. The hydrogen atoms had been omitted for clarity.

**Fig. 3:**
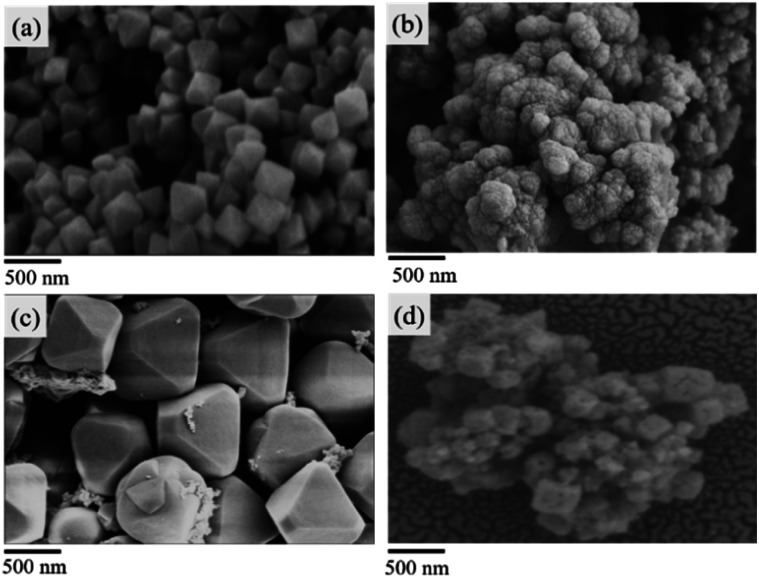
SEM pictures (a) Zr-UiO-66-NH_2_ modulated by 90 eq HAc and 1 eq HCl. (b) Zr-UiO-66-NH_2_ without acid modulation. (c) Zr-UiO-67-NH_2_ modulated by 90 eq HAc and 1 eq HCl. (d) Zr-UiO-67-NH_2_ without acid modulation.

The powder X-ray diffraction (PXRD) pattern of the acid-modulated Zr-UiO-66-NH_2_ metal–organic framework showed that almost all the main diffraction peaks were in accordance with the harmonic pattern of Zr-UiO-66-NH_2_ without any tuning (Fig. 1).^32^ The data show that the Zr_6_O_4_(OH)_4_ node was connected through the ligand carboxyl group, thereby establishing a framework with a cubic topology, and the basic structural model of the acid-regulated Zr-UiO-66-NH_2_ is unchanged (Fig. 2). However, the characteristic diffraction peak half width value indicated that the crystal size of the acid-modulated MOF was greater than the MOF without acid modulation. In addition, this result was verified on the basis of the SEM pictures, and the nano-crystals of Zr-UiO-66-NH_2_ and Zr-UiO-67-NH_2_ without acid modulation had smaller crystal structure (Fig. 3a and c). Moreover, the morphology of the crystals of Zr-UiO-66-NH_2_ modulated by acid was more prominent with a sharper edge and typical octahedral structure (Fig. 3b and d).

**Fig. 4:**
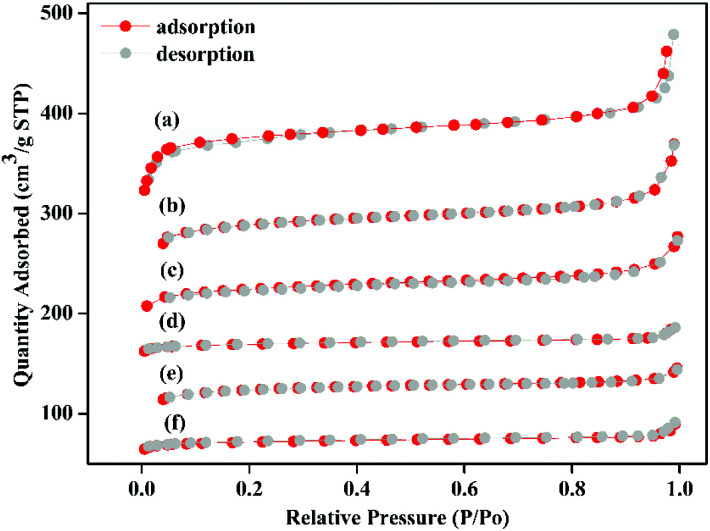
N_2_ adsorption–desorption isotherms. (a) Zr-UiO-67-NH_2_ modulated by 90 eq HAc and 1 eq HCl. (b) Zr-UiO-66-NH_2_ modulated by 90 eq HAc and 1 eq HCl. (c) Zr-UiO-66-NH_2_ modulated by 60 eq HAc and 1 eq HCl. (d) Zr-UiO-67-NH_2_ without acid modulation. (e) Zr-UiO-66-NH_2_ modulated by 30 eq HAc and 1 eq HCl. (f) Zr-UiO-66-NH_2_ without acid modulation.

The X-ray crystal structure confirms the properties of these materials, which were beneficial to the nitrogen adsorption–desorption isotherm experiment. It can be seen from Fig. 4, all samples exhibit typical I-type adsorption and desorption isotherms, indicating that the samples were all typical microporous structures. The structural parameters of the samples were shown in Table 1. The specific surface area of the Zr-UiO-66-NH_2_ sample synthesized by traditional method was 509 m^2^ g^−1^ and the pore diameter was 3.494 nm (Table 1f). And compared to traditional method, the nitrogen adsorption capacity of the MOF sample synthesized by acid modulation increased significantly, indicating an increase in porosity. The BET specific surface area of the sample increased to 800 m^2^ g^−1^ as the amount of acid added increases, and the pore diameter also showed an increasing trend from 3.494 nm to 7.565 nm (Table 1b). The increase in porosity may be due to the expansion of its ordered 3D network structure with increasing crystal size, which enriching the porosity. The increase of the pore diameter was beneficial to the later grafting and catalytic reactions. A similar trend occurred in the specific surface area of the Zr-UiO-66-NH_2_ sample (Table 1a and e), the pore diameter also showed an increasing trend from 5.236 nm to 9.235 nm. The BET experiments prove a series of UiO carriers with different pore sizes were synthesized.

**Table 1:** Specific surface area, pore volume and pore size^a^

Sample	BET [m^2^ g^−1^]	Volume [cm^3^ g^−1^]	Diameter [nm]
[a] Zr-UiO-67-NH_2_	1008	0.857	9.235
[b] Zr-UiO-66-NH_2_	800	0.503	7.565
[c] Zr-UiO-66-NH_2_	703	0.403	6.286
[d] Zr-UiO-67-NH_2_	656	0.369	5.236
[e] Zr-UiO-66-NH_2_	606	0.314	4.841
[f] Zr-UiO-66-NH_2_	509	0.205	3.494

a [a] Zr-UiO-67-NH_2_ modulated by 90 eq HAc and 1 eq HCl. [b] Zr-UiO-66-NH_2_ modulated by 90 eq HAc and 1 eq HCl. [c] Zr-UiO-66-NH_2_ modulated by 60 eq HAc and 1 eq HCl. [d] Zr-UiO-67-NH_2_ without acid modulation. [e] Zr-UiO-66-NH_2_ modulated by 30 eq HAc and 1 eq HCl. [f] Zr-UiO-66-NH_2_ without acid modulation.

**Fig. 5:**
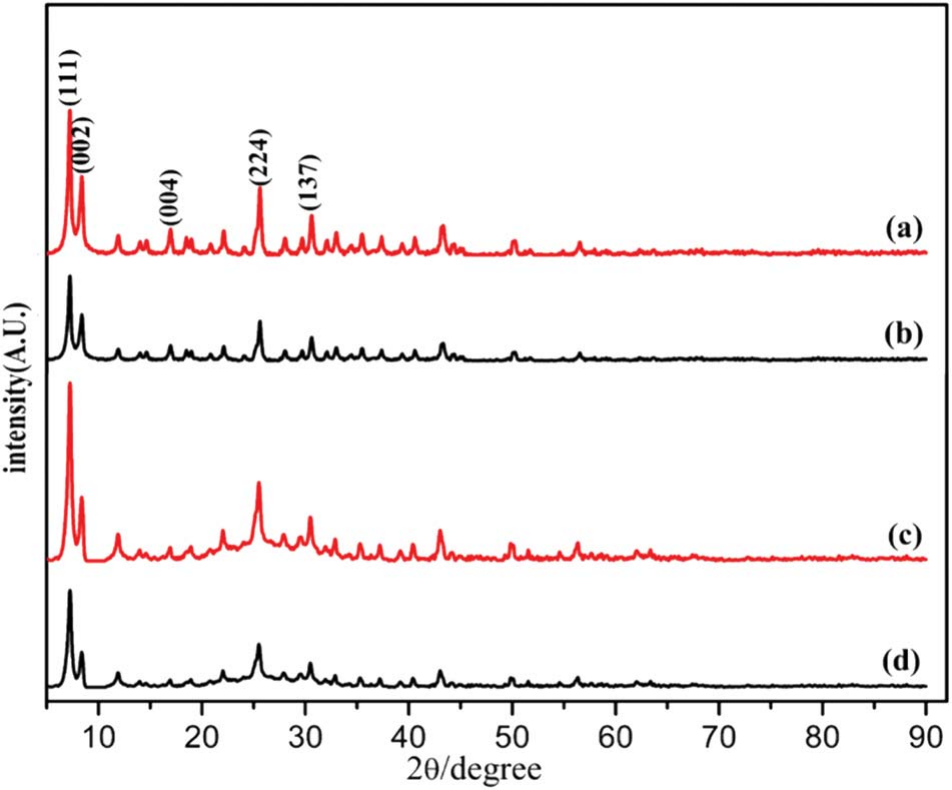
PXRD patterns of proline catalysts. (a) Zr-UiO-66-NH_2_ modulated by 90 eq HAc and 1 eq HCl, (b) Zr-UiO-66-NH_2_ without acid modulation, (c) Zr-UiO-67-NH_2_ modulated by 90 eq HAc and 1 eq HCl, (d) Zr-UiO-67-NH_2_ without acid modulation.

**Fig. 6:**
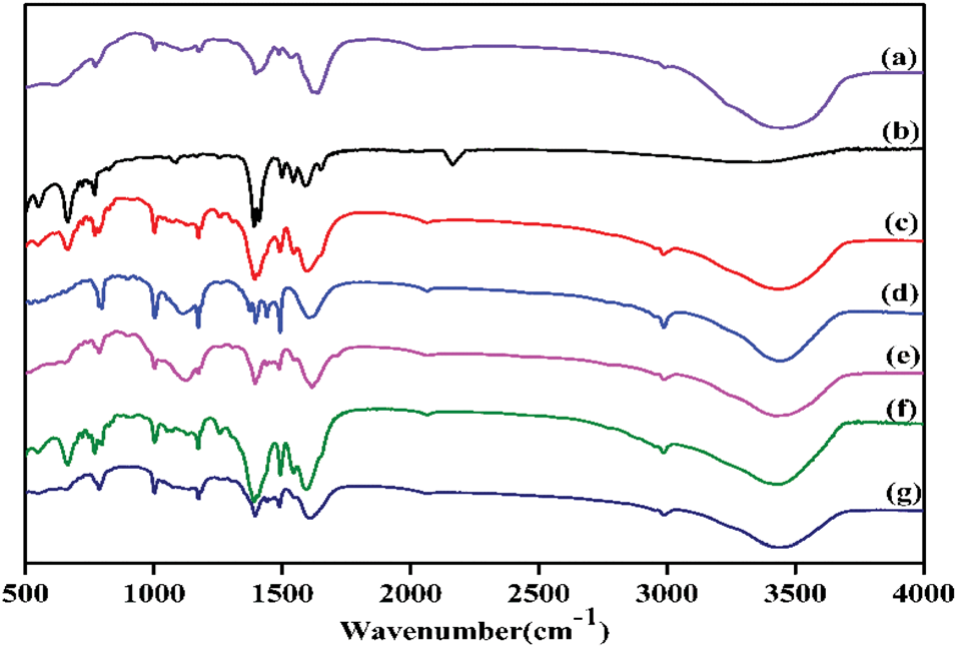
FT-IR of Zr-UiO-66-NH_2_ modified synthetic catalysts. (a) Zr-UiO-67-NHCS-Pro. (b) Zr-UiO-66-NCS. (c) Zr-UiO-66-NHCS-Leu. (d) Zr-UiO-66-NHCS-Ile. (e) Zr-UiO-66-NHCS-Lte. (f) Zr-UiO-66-NHCS-Trp. (g) Zr-UiO-66-NHCS-Pro.

PXRD of the as synthesized Zr-UiO-66-NHCS-NH(R) indicated the phrase purity of its bulky sample, where the diffraction pattern was similar to Zr-UiO-66-NH_2_. The results of PXRD pattern confirmed that solvent-free Zr-UiO-66-NHCS-NH(R), which was obtained by exchanging with dichloromethane and followed by drying under vacuum, retained the original skeleton of Zr-UiO-66-NH_2_ (Fig. 5 and S7†). The NMR spectrum of Zr-UiO-66-NH_2_ displays resonances at 6.97, 7.05 and 7.48 ppm which were characteristic of the three distinct protons on the amine functionalized aromatic ring of the 2-aminoterephthalic acid linker. In the case of the postmodified MOFs, a new set of peaks was observed in the NMR spectra of Zr-UiO-66-NHCS-Leu, Zr-UiO-66-NHCS-Ile, Zr-UiO-66-NHCS-Lte, Zr-UiO-66-NHCS-Trp, and Zr-UiO-66-NHCS-Pro (Fig. S1–S5†). Comparison of peak area of the characteristic peak allows the grafting rate for each post-synthetic modification reaction to be calculated. Furthermore, IR analysis was conducted to prove the structure of UiO-66-NHCS-NH(R). The characteristic stretching vibration peak at 2164 cm^−1^ conformed to the existence of isothiocyanate (*ν*_N=C=S_), and it disappeared in the IR of MOF–NHCS-Pro when the stretching vibration peak appeared at (*ν*_N–C=S(I)_) 1003 cm^−1^, (*ν*_N–C=S(II)_) 1087 cm^−1^ and (*ν*_N–C=S(III)_) 1396 cm^−1^ could indicate that the successful grafting of amino acid derivatives (Fig. 6).

### Catalysis and cycling experiment

The activated Zr-MOF–NHCS–NH(R) was directly applied to catalyse the asymmetric aldol reaction of 4-nitrobenzaldehyde and acetone. As shown in Table 2, using a certain amount of acid modulator in the synthesis of Zr-UiO-66-NH_2_, relevant Zr-UiO-66-NHCS-Pro catalyses the reaction to provide the aldol product 4-hydroxy-4-(4-nitrophenyl)-butan-2-one with 73% ee and 83% yield (Table 2, entry 4), whereas the skeleton of Zr-UiO-66-NH_2_ without adding acid shows an ee of only 26% and a yield of 30% (Table 2, entry 1). With the increase of the pore size of catalyst carrier, the yield and ee-value basically increased (Table 2, entries 1–4 and 10). But the enantioselectivity decreased to 50% when the hole size was further increased by the addition of acid in the synthesis of UiO-67-NH_2_ (Table 2, entry 9). These results indicate that the performance of the heterogeneous catalyst may be improved by controlling of the aperture of MOF cavities and the MOF-based amino acid–thiourea catalysis could occur at substantially higher rates and enantioselectivity, even be close to homogeneous catalysis (Table 2, entry 15). However, the catalytic efficiency of the catalytic derivatives from other amino acids is low (Table 2, entries 5–8). It may be affected greatly by the rigid ring frame of the 2° amino. Compared with the reported experimental results (Table 2, entries 9–12), the catalytic effect of Zr-UiO-66-NHCS-Pro has some certain advantages.

**Table 2:** Screening of catalyst^a^

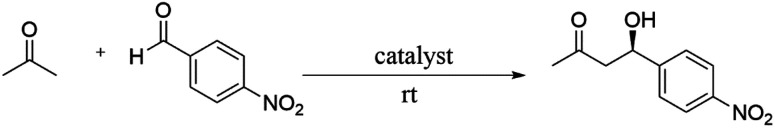					
Entry	Catalyst	Time (h)	Yield^b^ (%)	ee^c^ (%)	Ref
1	Zr-UiO-66-NHCS-Pro^d^	48	30	26	This work
2	Zr-UiO-66-NHCS-Pro^e^	48	52	35	This work
3	Zr-UiO-66-NHCS-Pro^f^	48	69	59	This work
4	Zr-UiO-66-NHCS-Pro^g^	48	83	73	This work
5	Zr-UiO-66-NHCS-Leu	48	32	9	This work
6	Zr-UiO-66-NHCS-Ile	48	34	12	This work
7	Zr-UiO-66-NHCS-Lte	48	36	18	This work
8	Zr-UiO-66-NHCS-Trp	48	38	23	This work
9	Zr-UiO-67-NHCS-Pro^g^	24	85	50	This work
10	Zr-UiO-67-NHCS-Pro^d^	24	80	65	This work
11	IRMOF-Pro	40	>95^h^	29	Ref. 34
12	Al-MIL-101-NH-Pro	168	18	18	Ref. 35
13	Al-MIL-101-NH-Gly-Pro	168	80^h^	27	Ref. 35
14	Zn-MOF-74-L-Pro	20	4	15	Ref. 36
15	CMIL-1	36	66	69	Ref. 37
16	IRMOF-3-Pr(OP)	72	11	89	Ref. 33
17	Proline	4	68	76	Ref. 38

a Reaction performed using 20 mol% of catalyst (0.05 mmol of proline derivative in MOF), *p*-nitro-benzaldehyde (0.2 mmol), in acetone (2 mL) at room temperature.

b Yield of the isolated adducts.

c Determined by HPLC.

d No acid was added in the synthesis of Zr-MOF-NH_2_.

e 30 eq HAc, 1 eq HCl were added in the synthesis of Zr-UiO-66-NH_2_.

f 60 eq HAc, 1 eq HCl were added in the synthesis of Zr-UiO-66-NH_2_.

g 90 eq HAc, 1 eq HCl were added in the synthesis of Zr-MOF-NH_2_.

h The use of 100 mol% of catalyst.

The effects of nine different polar solvents on the catalytic activity of the catalysts were investigated. The results were shown in Table 3. The product yields (18% to 83%) and corresponding ee-values (0% to 73%) changed significantly with the change of solvent polarity. Experiments show that acetone is the best solvent of this reaction. This is not only affected by the polarity of the solvent, but also may be related to the solvent adsorption and desorption process in heterogeneous catalysts. Therefore, acetone was used as the catalytic reaction solvent in subsequent experiments.

**Table 3:** Influence of solvents a

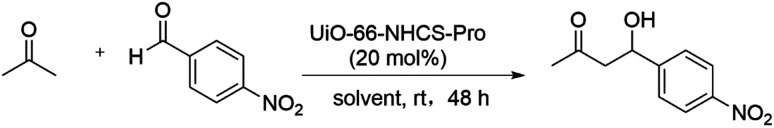			
Entry	Solvent	Yield^b^ (%)	ee^c^ (%)
1	THF	25	32
2	Toluene	33	35
3	H_2_O	46	45
4	CHCl_3_	28	2
5	CH_3_CN	30	5
6	CH_3_OH	28	11
7	C_2_H_5_OH	31	8
8	CH_2_Cl_2_	18	0
9	Acetone	83	73

a Reaction performed using 20 mol% of catalyst (0.05 mmol of proline derivative in MOF), *p*-nitro-benzaldehyde (0.2 mmol) and acetone (2 mmol), in solvent (2 mL) at room temperature.

b Yield of the isolated adducts.

c Determined by HPLC.

Using Zr-UiO-66-NHCS-Pro as catalyst, the effect of catalyst dosage on the catalytic reaction was investigated. As shown in the Table 4, increasing the amount of catalyst from 20 mol% to 30 mol% under the same conditions could not further improve the ee-value, but the yield slightly increased up to 92% (Table 4, entry 4). When the catalyst dosage was reduced to 5 mol%, the catalytic rate and ee-value decreased evidently (Table 4, entry 1).

**Table 4:** Effect of dosage of Zr-UiO-66-NHCS-Pro catalyst on asymmetric aldol reaction^a^

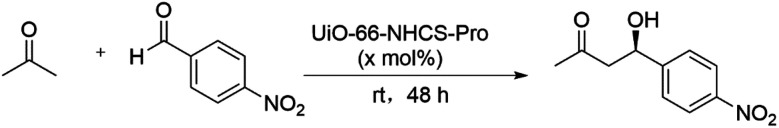			
Entry	mol%	Yield^b^ (%)	ee^c^ (%)
1	5	71	50
2	10	70	65
3	20	83	73
4	30	92	68

a Reaction performed using 5–30 mol% of catalyst, *p*-nitro-benzaldehyde (0.2 mmol) in acetone (2 mL) at room temperature.

b Yield of the isolated adducts.

c Determined by HPLC.

**Fig. 7:**
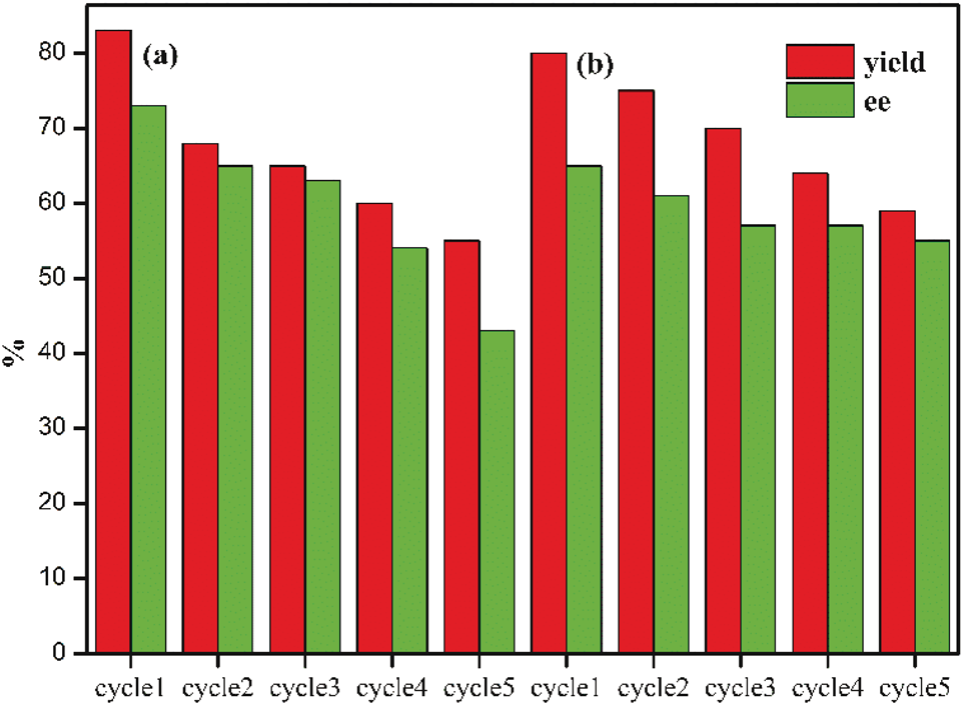
Investigating the cycling performance of the catalyst. (a) Zr-UiO-66-NHCS-Pro, (b) Zr-UiO-67-NHCS-Pro.

**Fig. 8:**
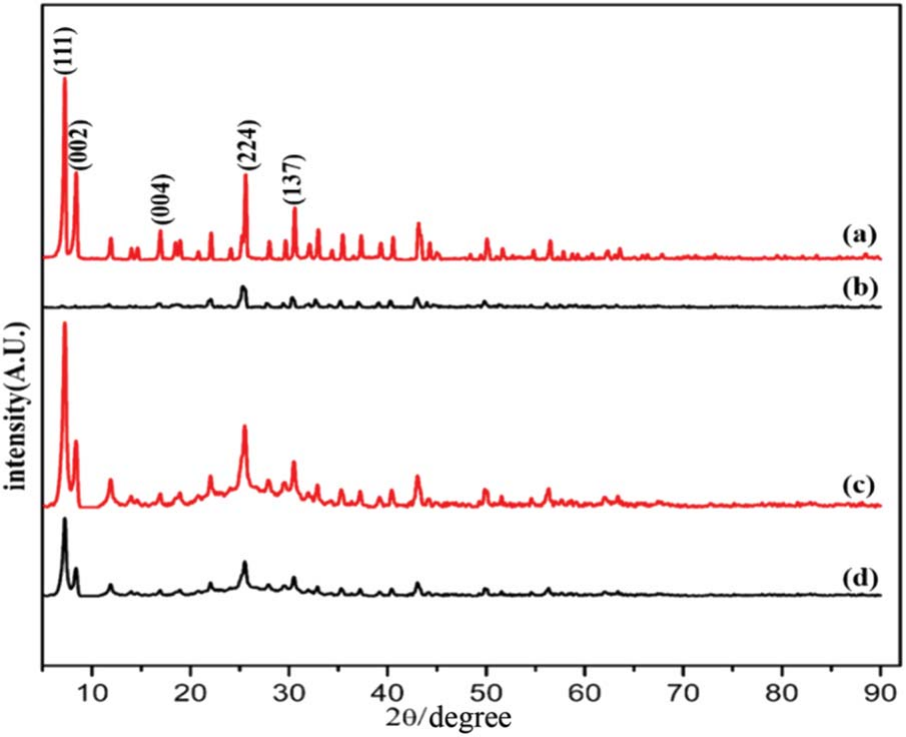
PXRD of before and after catalyst reaction. (a) Zr-UiO-66-NHCS-Pro before the reaction. (b) Zr-UiO-66-NHCS-Pro after three cycles. (c) Zr-UiO-67-NHCS-Pro before the reaction. (d) Zr-UiO-66-NHCS-Pro after five cycles.

**Fig. 9:**
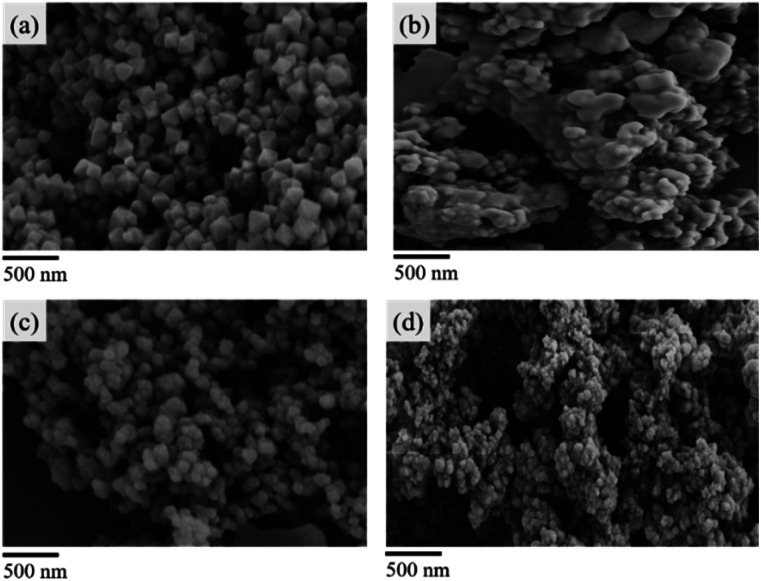
SEM pictures of the catalyst. (a) Zr-UiO-66-NHCS-Pro before the reaction. (b) Zr-UiO-66-NHCS-Pro after three cycles. (c) Zr-UiO-67-NHCS-Pro before the reaction. (d) Zr-UiO-66-NHCS-Pro after five cycles.

In testing the recyclability of the Zr-UiO-66-NHCS-Pro catalyst, five cycles of aldol reaction were performed. The solid catalyst was separated by centrifugation from the suspension after 48 h, washed with CH_2_Cl_2_, and dried under vacuum for recycling. As shown in (Fig. 7a), catalytic reaction by Zr-UiO-66-NHCS-Pro show a gradual decrease in ee-value after recycling. In particular, the ee-value has decreased below 60% after three cycles, and the yield also gradually decreases. Compared with the original catalyst XRD (Fig. 8a and b), the characteristic peaks of XRD weakened and gradually disappeared after the third cycle, indicating that the crystal crystallinity of MOF changed. And the disappearance of the typical octahedral structures in the SEM images of the catalyst (Fig. 9b) indicated that the catalyst support skeleton may gradually be destroyed under the vigorous stirring in the reaction. The decreased stability of the MOF may be due to increased crystal defects caused by acid regulation. And the reaction environment in the MOF cavities has been changed a lot. It could be the chief culprit for the decline of the enantioselectivity.

Considering the degradation of structural stability may be caused by acid regulation, the UiO-67-NHCS-Pro was reused for five cycles of aldol reaction (Fig. 7b). By the same methods, reactions catalysed by Zr-UiO-67-NHCS-Pro also show a gradual decrease in ee-value and yield after recycling. But the ee-value has remained the same after three cycles. The PXRD (Fig. 7c and d) and the SEM images (Fig. 9c and d) of the catalyst indicated that the basic skeleton and crystal crystallinity remained after five cycles. It confirmed the important influence of MOF skeletal and pore structure on the catalytic performance of catalysts from the side.

## Conclusions

Herein, we reported an efficient and easily synthesis method for grafting amino acid-derived chiral moieties inside MOF cavities. By incorporating acidic modulators to control the cavity size of Zr-UiO-66-NH_2_. A series of Zr-UiO-66-NHCS-Leu, Zr-UiO-66-NHCS-Ile, Zr-UiO-66-NHCS-Lte, Zr-UiO-66-NHCS-Trp, Zr-UiO-66-NHCS-Pro and Zr-UiO-67-NHCS-Pro were obtained by post-synthesis modification. Among them, the amino acid–thiourea motif was first anchored in the MOF cavities with high yield. And the thiourea group formed by mild reaction conditions from the high reactive isothiocyanate can avoid racemization of chiral skeletons in synthesis and provide hydrogen bond donors which can effectively improve the catalytic activity and enantioselectivity. We found that the Zr-UiO-66-NHCS-Pro catalyst has well catalytic activity for catalysing the asymmetric aldol reaction between 4-nitrobenzaldehyde and acetone in good yields and enantioselectivities. Meanwhile, the important effect of a suitable reaction environment by controlling the aperture of MOF cavities for the activity and selectivity of heterogeneous catalysts was confirmed by a series of experiments on catalyst MOF carriers with different pore sizes.

## Author contributions

J. F. Zhu designed the schemed and performed the experiments; J. F. Zhu and W. Liu wrote the paper; X. R. Meng and W. Liu contributed to the supervision and revising; all authors contributed to the general discussion and the experiments.

## Conflicts of interest

There are no conflicts to declare.

## Supplementary Material

RA-012-D2RA03747E-s001
