# Supplementary material for: Regulated synthesis of Zr-metal–organic frameworks with variable hole size and its influence on the performance of novel MOF-based heterogeneous amino acid–thiourea catalysts
Source: RSC Adv. 2022 Aug 3;12(33):21574–81. doi: 10.1039/d2ra03747e (PMC9347210; doi:10.1039/d2ra03747e)
Supplement: RA-012-D2RA03747E-s001 [file RA-012-D2RA03747E-s001.pdf]

Electronic Supplementary Material (ESI) for RSC Advances.
This journal is © The Royal Society of Chemistry 2022

Electronic Supplementary Material (ESI) for RSC Advances.
This journal is © The Royal Society of Chemistry 2022

Supplementary information

Regulated synthesis of Zr-metal-organic frameworks with variable hole size and its influence on the performance of Novel MOF-based heterogeneous amino acid-thiourea catalyst

Junfeng Zhu,[a] Xiaorong Meng,*[a] Wen Liu,*[a] Yabing Qi,[a] Siyi Jin [a] and Shanshan Huo [b]

a College of Chemistry and Chemical Engineering, Xi'an University of Architecture and Technology, Xi'an 710055, PR China.
b Research Institute of Membrane Separation Technology of Shaanxi Province Co., Ltd, Xi'an 710055, China.

∗ Corresponding author. E-mail addresses: mxr5@163.com (Xiaorong Meng); 24456132@qq.com (Wen Liu)

Experiment section

Synthesis and characterization of catalysts

1. Synthesis of Zr-UiO-66-NHCS-Leu

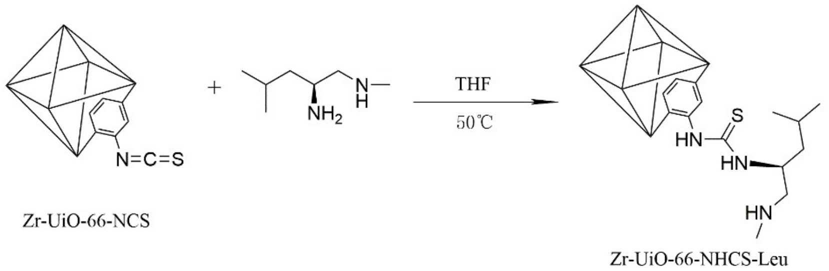

In a 50 mL round-bottomed flask, 1.20 mmol of 2-amino-4-methylpentylmethylamine was dispersed in 20 mL of anhydrous tetrahydrofuran, and the required amount of Zr-UiO-66-NH_2 was added (1.00 mmol). The solution was stirred at 50°C for 24 h. Then, it was centrifuged, and the solid was washed with THF (3×10 mL) to obtain as a fine yellow powder after drying in vacuo at 50°C.

^{1}H NMR (500 MHz, Deuterium Oxide) δ 8.26 (d, J = 1.5 Hz, 1H), 7.95 (dd, J = 7.8, 1.5 Hz, 1H), 7.33 (d, J = 7.8 Hz, 1H), 3.52-3.49 (m, 5H), 2.03 (s, 3H), 1.71 – 1.53 (m, 6H), 0.66-0.62 (m, 1H).

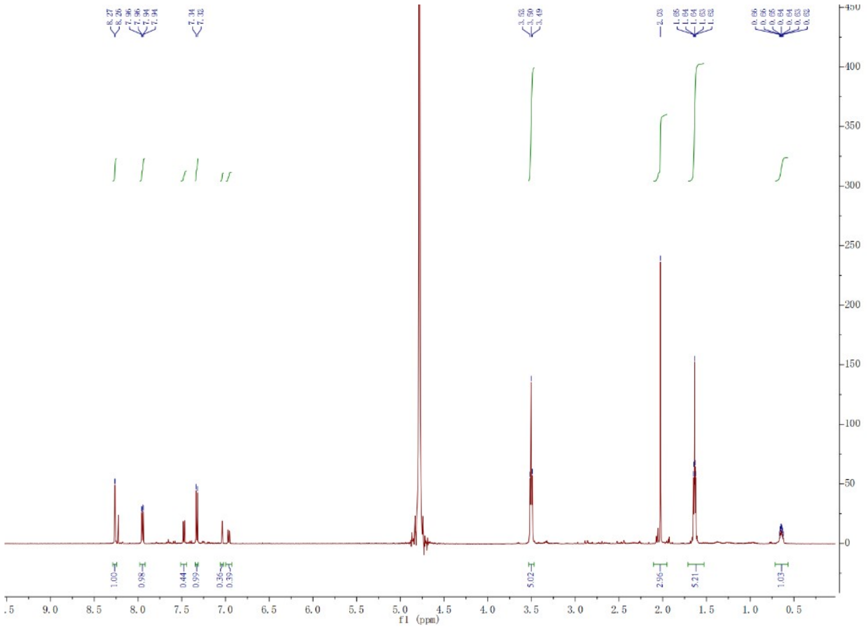

Fig. S1 Representative ^{1}H NMR spectrum of Zr-UiO-66-NHCS-Leu dissolved in NaOH-D_2O solution (ca. 64% modified). Unmodified Zr-UiO-66-NH_2 and functionalized linker were identified at a peak area of 6.9–8.5 ppm.

2. Synthesis of Zr-UiO-66-NHCS-Ile

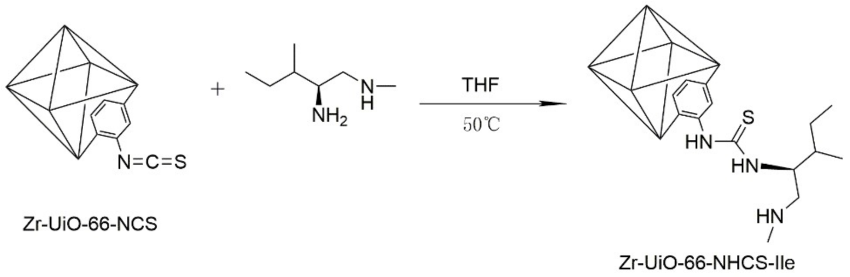

In a 50 mL round-bottomed flask, 1.20 mmol of 3-amino-4-methylpentylmethylamine was dispersed in 20 mL of anhydrous tetrahydrofuran, and add the required amount of Zr-UiO-66-NH_2 was added (1.00 mmol). The

solution was stirred at 50°C for 24 h. Then, it was centrifuged, and the solid was washed with THF (3×10 mL) to obtain as a fine yellow powder after drying in vacuo at 50°C.

^{1}H NMR (500 MHz, Deuterium Oxide) δ 7.79 (s, 1H), 7.48 (d, J = 7.8 Hz, 1H), 6.86 (d, J = 7.8 Hz, 1H), 3.04 (m, 3H), 2.09 (s, 1H), 1.56 (s, 3H), 1.17 (m, 3H).

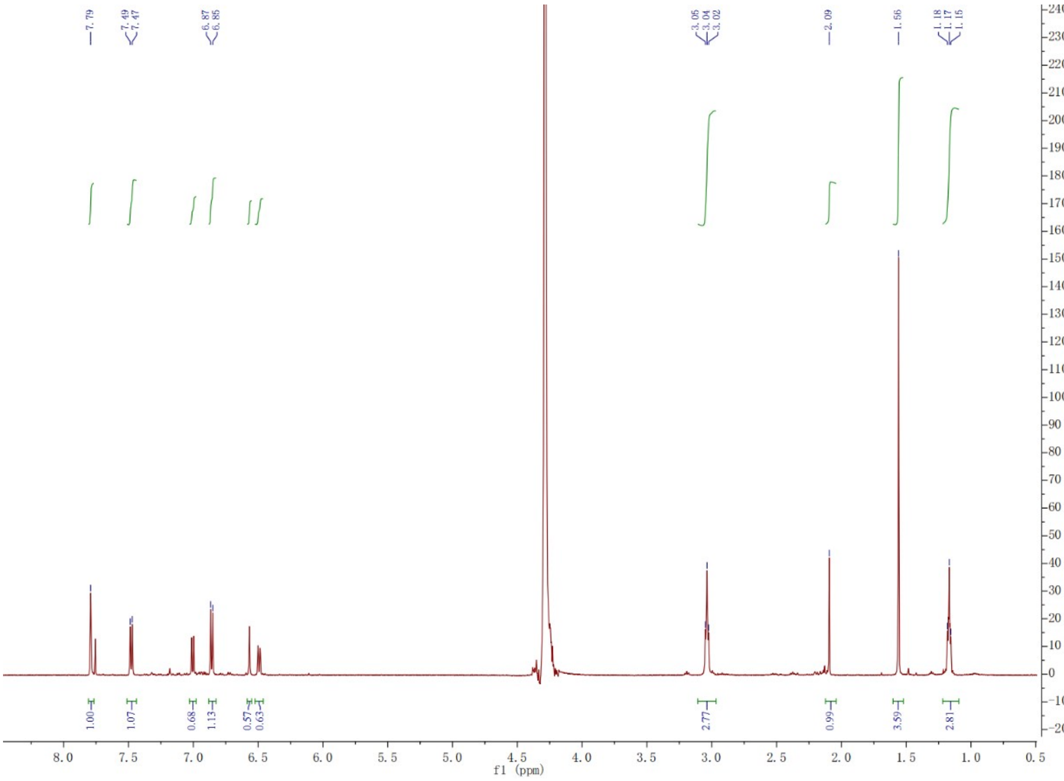

Fig. S2 Representative ^{1}H NMR spectrum of Zr-UiO-66-NHCS-Ile dissolved in NaOH-D_2O solution (ca. 43% modified). Unmodified Zr-UiO-66-NH_2 and functionalized linker were identified at a peak area of 6.8-8.0 ppm.

3. Synthesis of Zr-UiO-66-NHCS-Lte

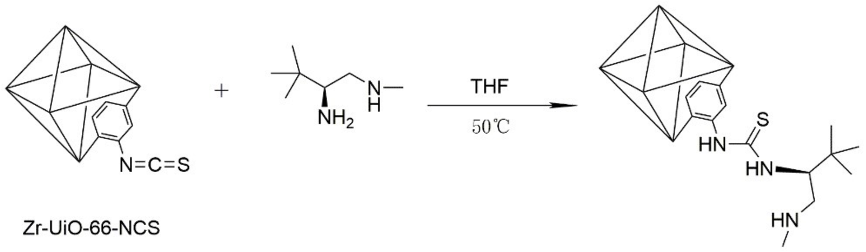

Zr-UiO-66-NCS

Zr-UiO-66-NHCS-Lte

In a 50 mL round-bottomed flask, 1.20 mmol of 2-amino-3,3-dimethylbutylmethylamine was dispersed in 20 mL of anhydrous tetrahydrofuran, and the required amount of Zr-UiO-66-NH_2 was added (1.00 mmol). The solution was stirred at 50°C for 24 h. Then, it was centrifuged, and the solid was washed with THF (3×10 mL) to obtain as a fine yellow powder after drying in vacuo at 50°C.

^{1}H NMR (500 MHz, Deuterium Oxide) δ 8.33 (d, J = 1.4 Hz, 1H), 8.01 (dd, J = 7.8, 1.4 Hz, 1H), 7.40 (d, J = 7.8 Hz, 1H), 2.69 (s, 2H), 2.52 (s, 1H), 2.10 (m, 12H).

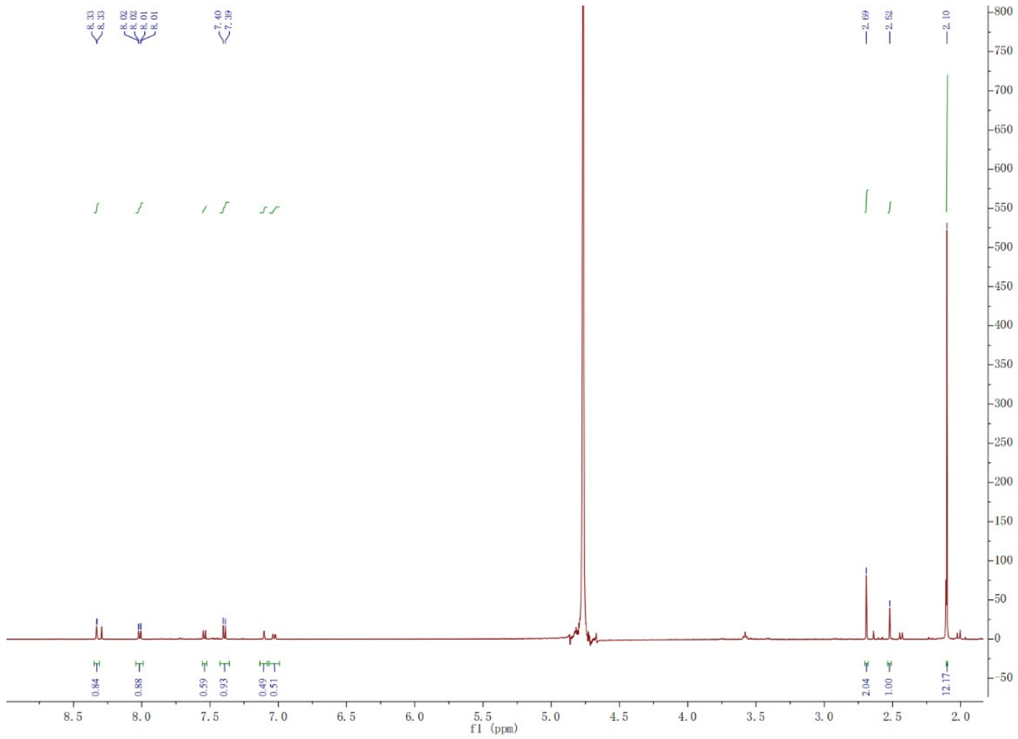

Fig. S3 Representative ^{1}H NMR spectrum of Zr-UiO-66-NHCS-Lte dissolved in NaOH-D_2O solution (ca. 42% modified). Unmodified Zr-UiO-66-NH_2 and functionalized linker were identified at a peak area of 6.9–8.5 ppm.

4. Synthesis of Zr-UiO-66-NHCS-Trp

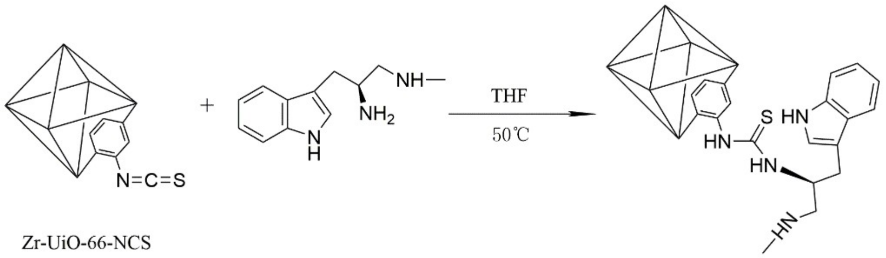

Zr-UiO-66-NCS

Zr-UiO-66-NHCS-Trp

In a 50 mL round-bottomed flask, 1.20 mmol of α-aminoindolylpropylmethylamine was dispersed in 20 mL of anhydrous tetrahydrofuran, and the required amount of Zr-UiO-66-NH_2 was added (1.00 mmol). The solution was stirred at 50°C for 24 h. Then it was centrifuged, and the solid was washed with THF (3×10 mL) to obtain as a fine yellow powder after drying in vacuo at 50°C.

^{1}H NMR (500 MHz, Chloroform-d) δ 8.33 (s, 1H), 7.64 (d, J = 7.8 Hz, 1H), 7.37 (d, J = 8.1 Hz, 1H), 7.26 (s, 1H), 7.19 (t, J = 8.0 Hz, 1H), 7.12 (t, J = 7.5 Hz, 1H), 7.08 – 6.99 (m, 1H), 5.78 (s, 1H), 5.18 (s, 1H), 4.40 (s, 1H), 3.31 (m, 1H), 3.19 – 3.10 (m, 1H), 2.96 (s, 1H), 2.81 (s, 3H), 2.67-2.65 (m, 3H).

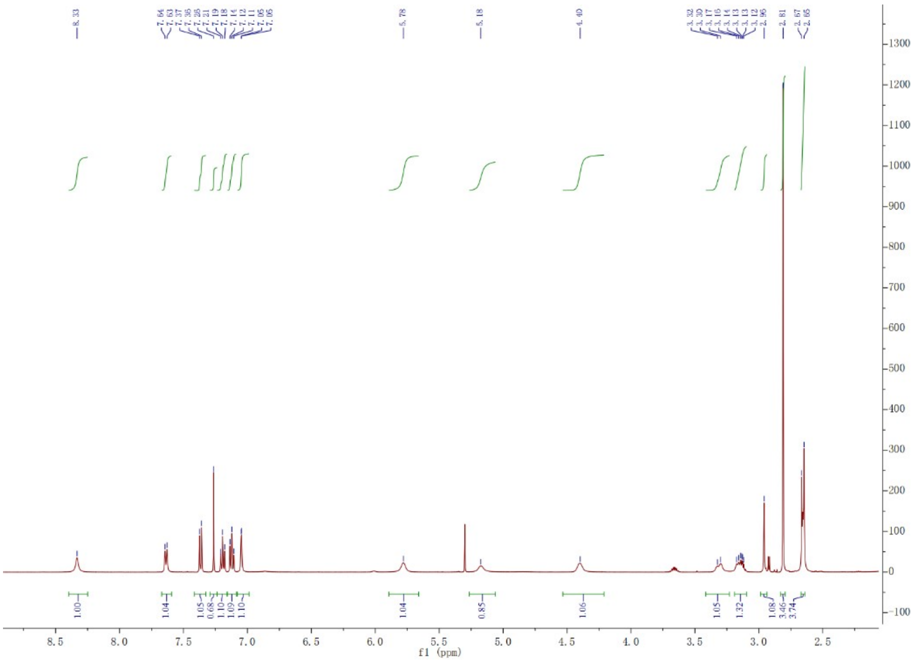

Fig. S4 Representative ^{1}H NMR spectrum of Zr-UiO-66-NHCS-Trp dissolved in NaOH-D_2O solution (ca. 50% modified). Unmodified Zr-UiO-66-NH_2 and functionalized linker were identified at a peak area of 7.0–8.5 ppm.

5. Synthesis of Zr-UiO-66-NHCS-Pro

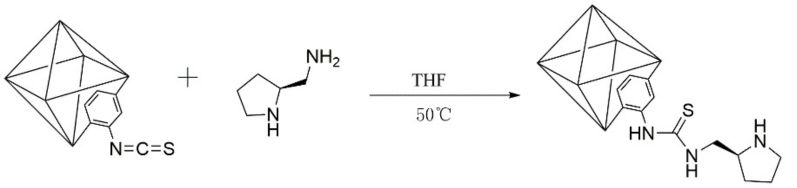

Zr-UiO-66-NCS

Zr-UiO-66-NHCS-Pro

In a 50 mL round-bottomed flask, 1.20 mmol of Pyrrolidine-2-methylamine was dispersed in 20 mL of anhydrous tetrahydrofuran, and the required amount of Zr-UiO-66-NH_2 was added (1.00 mmol). The solution was stirred at 50°C for 24 h. Then, it was then centrifuged, and the solid was washed with THF (3×10 mL) to obtain as a fine yellow powder after drying in vacuo at 50°C.

^{1}H NMR (500 MHz, Deuterium Oxide) δ 8.35 (s, 1H), 8.03 (d, J = 7.7 Hz, 1H), 7.43 – 7.35 (m, 1H), 2.89 – 2.77 (m, 1H), 2.69 – 2.54 (m, 2H), 2.48 – 2.35 (m, 1H), 1.94-1.93 (m, 1H), 1.77-1.70 (m, 1H), 1.60 – 1.51 (m, 3H).

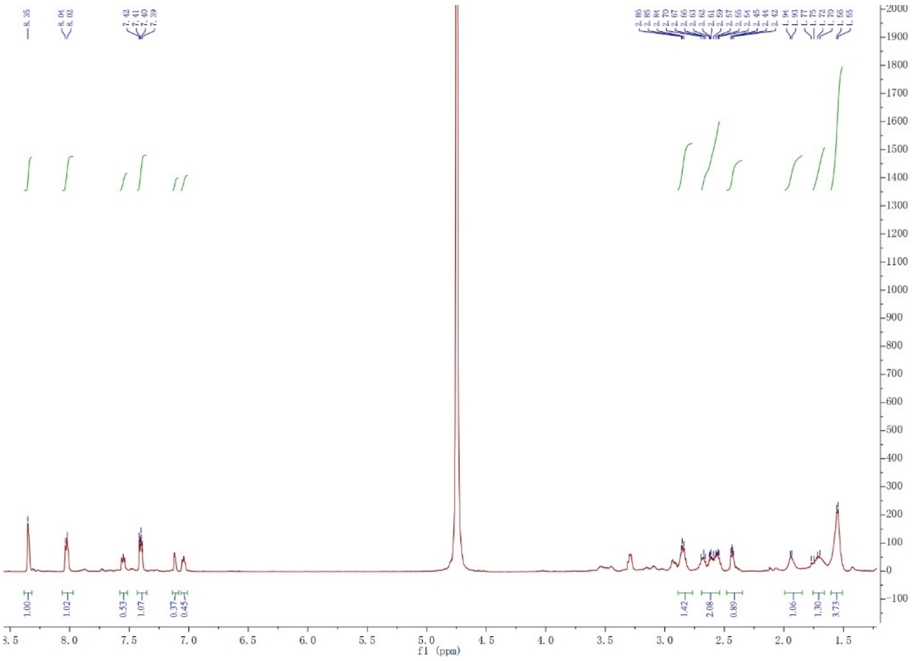

Fig. S5 Representative ^1H NMR spectrum of Zr-UiO-66-NHCS-Pro dissolved in NaOH-D_2O solution (ca. 67 % modified). Unmodified Zr-UiO-66-NH_2 and functionalized linker were identified at a peak area of 7.0–8.5 ppm.

6. Synthesis of Zr-UiO-67-NHCS-Pro

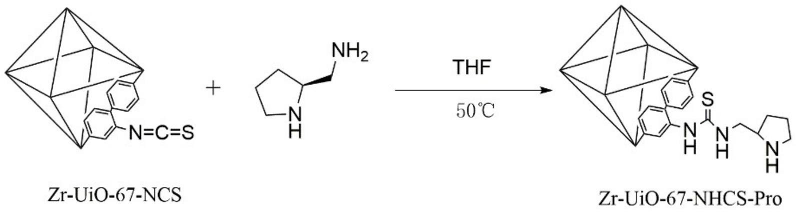

In a 50 mL round-bottomed flask, 1.20 mmol of Pyrrolidine-2-methylamine was dispersed in 20 mL of anhydrous tetrahydrofuran, and the required amount of Zr-UiO-67-NH_2 was added (1.00 mmol). The solution was stirred at 50°C for 24 h. Then, it was then centrifuged, and the solid was washed with THF (3×10 mL) to obtain as a fine yellow powder after drying in vacuo at 50°C.

^1H NMR (500 MHz, Deuterium Oxide) δ 7.74-7.72 (m, 3H), 7.52 (d, *J* = 8.3 Hz, 2H), 7.19 – 7.15 (m, 1H), 2.78-2.72 (m, 1H), 2.63 – 2.47 (m, 2H), 2.35-2.32 (m, 2H), 2.03 (s, 1H), 1.65-1.60 (m, 1H), 1.51 – 1.40 (m, 2H), 1.11 – 0.99 (m, 1H).

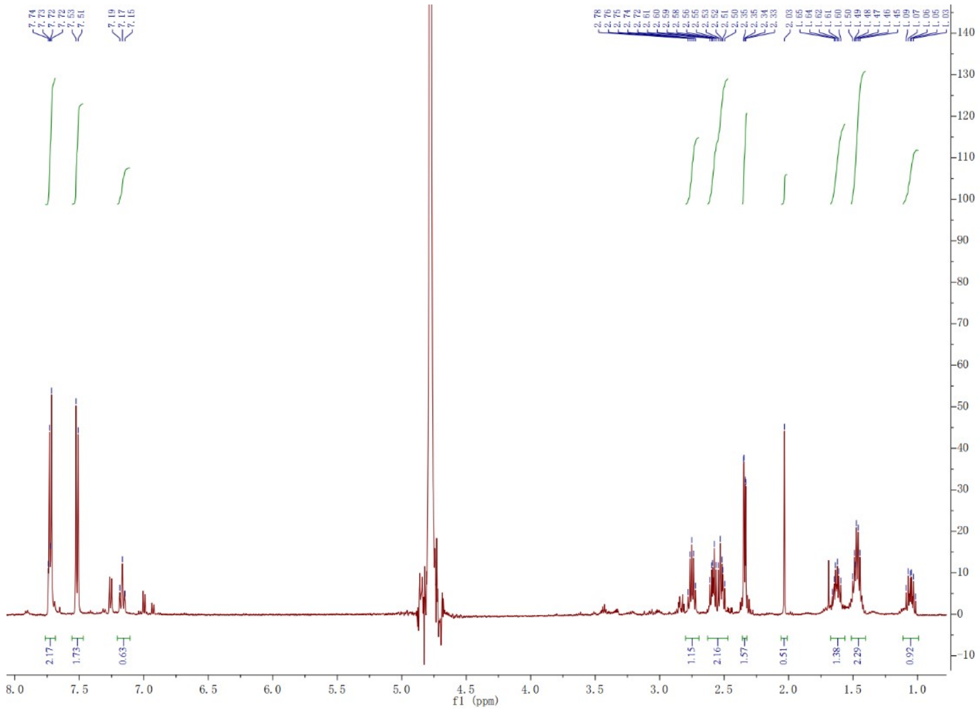

Fig. S6 Representative ^{1}H NMR spectrum of Zr-UiO-67-NHCS-Pro dissolved in NaOH-D_2O solution (ca. 63 % modified). Unmodified Zr-UiO-67-NH_2 and functionalized linker were identified at a peak area of 7.0–8.0 ppm.

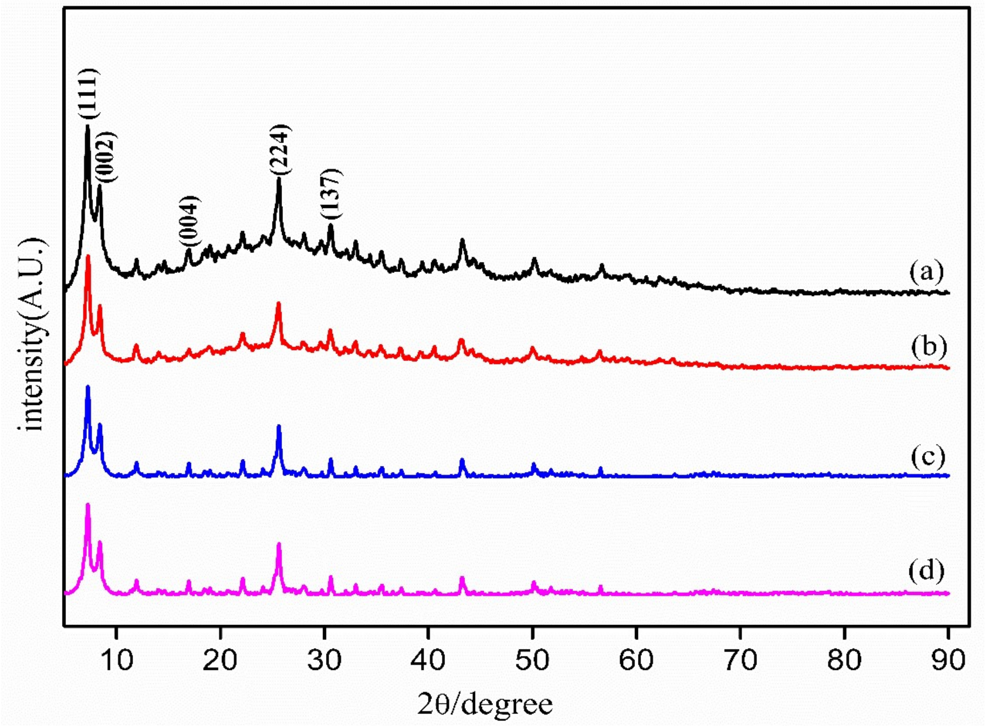

Fig. S7 PXRD patterns of different catalysts. (a) Zr-UiO-66-NHCS-Leu. (b) Zr-UiO-66-NHCS-Ile. (d) Zr-UiO-66-NHCS-Lte. (c) Zr-UiO-66-NHCS-Trp. (d) Zr-UiO-66-NHCS-Pro.

Catalysis of aldol reaction

$$ee=\frac{Area_{max}-Area_{min}}{Area_{max}+Area_{min}}$$

In a typical catalytic run, 30 mg of Zr-UiO-66-NHCS-Pro which corresponds to 0.05 mmol of proline moiety, was suspended in a solution of p-nitro-benzaldehyde (30 mg, 0.20 mmol) in acetone (1 mL). The suspension was allowed to react at room temperature and monitored by thin layer chromatography (TLC). After two days, the p-nitro-benzaldehyde was vanished from TLC. Then, the suspension was centrifuged. The solid catalyst was washed by CH_2Cl_2 (3×1 mL); the organic phases were combined and separated by TLC. Then, after concentration, a pale-yellow solid was obtained, ^{1}H NMR (500 MHz, Chloroform-*d*) δ 8.21 (d, *J* = 8.8 Hz, 2H), 7.54 (d, *J* = 8.8 Hz, 2H), 5.27 (m, 1H), 2.86 (m, 2H), 2.23 (s, 3H) and it was analyzed by HPLC (AS-H column, hexane: isopropanol = 70:30, 1 mL/min, 254 nm) to obtain enantiomeric excess (ee).

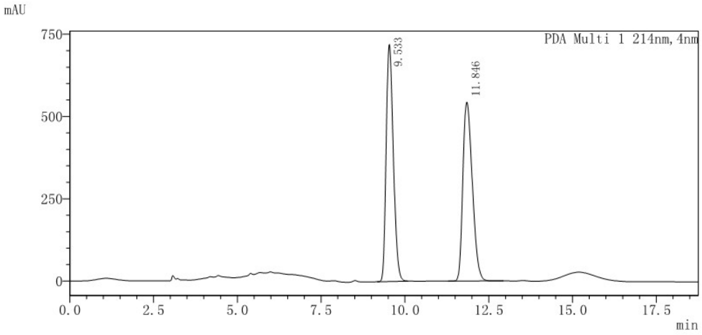

〈峰表〉
PDA Ch1 214nm

保留时间	面积	浓度	高度	面积%
9.533	10706077	0.000	720439	50.066
11.846	10677852	0.000	543200	49.934
	21383929		1263639	100.000

Fig. S8 HPLC of racemic modification.

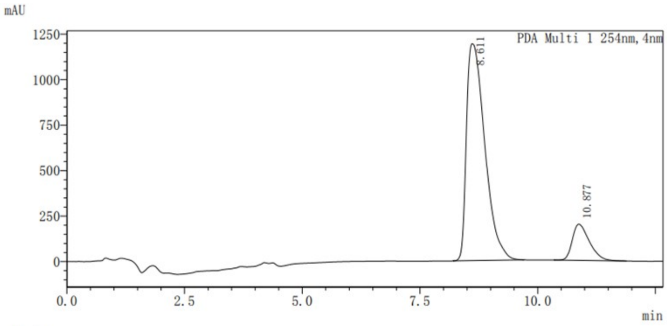

〈峰表〉
PDA Ch1 254nm

峰号	保留时间	面积	高度	浓度	浓度单位	标记	化合物名
1	8.611	32334423	1192334	0.000		M	
2	10.877	5023603	199209	0.000		M	
总计		37358025	1391543				

Fig. S9 Typical HPLC trace for the aldol reaction catalyzed by Zr-UiO-66-NHCS-Pro (cycle 1, 83% yield, 73% ee).

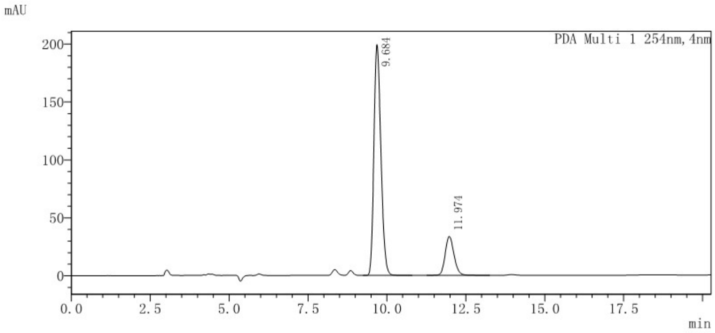

〈峰表〉
PDA Ch1 254nm

保留时间	面积	浓度	高度	面积%
9.684	3076140	0.000	199311	82.534
11.974	650995	0.000	33518	17.466
	3727136		232830	100.000

Fig. S10 Typical HPLC trace for the aldol reaction catalyzed by Zr-UiO-66-NHCS-Pro (cycle 2, acetone, 68% yield, 65% ee).

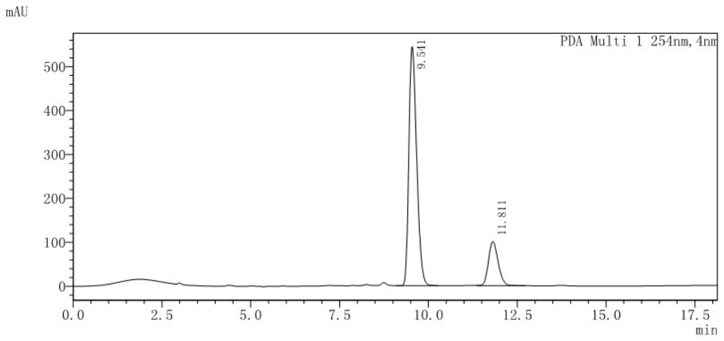

〈峰表〉
PDA Ch1 254nm

保留时间	面积	浓度	高度	面积%
9.541	8329137	0.000	544451	81.454
11.811	1896427	0.000	100246	18.546
	10225563		644696	100.000

Fig. S11 Typical HPLC trace for the aldol reaction catalyzed by Zr-UiO-66-NHCS-Pro (cycle 3, 65% yield, 63% ee).

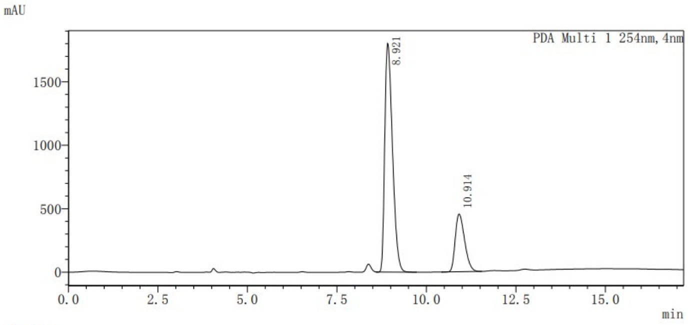

〈峰表〉
PDA Ch1 254nm

峰号	保留时间	面积	高度	浓度	浓度单位	标记	化合物名
1	8.921	27021724	1800593	0.000		M	
2	10.914	8306958	455464	0.000		M	
总计		35328681	2256057				

Fig. S12 Typical HPLC trace for the aldol reaction catalyzed by Zr-UiO-66-NHCS-Pro (cycle 4, 60% yield, 54% ee).

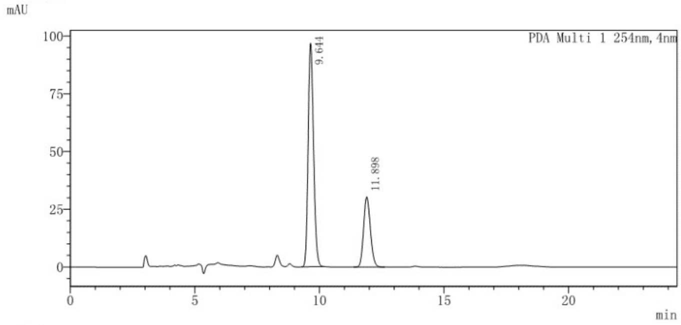

〈峰表〉
PDA Ch1 254nm

保留时间	面积	浓度	高度	面积%
9.644	1452475	0.000	96797	71.677
11.898	573936	0.000	30324	28.323
	2026411		127121	100.000

Fig. S13 Typical HPLC trace for the aldol reaction catalyzed by Zr-UiO-66-NHCS-Pro (cycle 5, 55% yield, 43% ee).
